# An Account of a Remarkable Fact Relative to the Small Pox

**Published:** 1786

**Authors:** William Wright

**Affiliations:** Fellow of the Royal College of Physicians at Edinburgh, and one of the Physicians General in Jamaica


XIV. *An Account of a remarkable Fact relative to the Small Pox. Communicated in a Letter to Dr.* Simmons, *F. R. S. by* William Wright, *M. D. F. R. S. Fellow of the Royal College*

*of Physicians at Edinburgh, and one of the Physicians General in Jamaica.*

HAVING lately read an account of a curious fact relative to inoculation, communicated by Mr. Dawson *, surgeon at Sedbergh, in Yorkshire, to the College of Physicians in London, and published in the third volume of their Transactions; I beg leave to observe to you, that in the course of a long and extensive country practice in Jamaica, many facts have occurred to convince me, that in the case of the small pox, a person may have a local affection without the habit in general being tainted by the variolous poison. I have often had occasion to observe, that the arms of patients inoculated will inflame and discharge an ichor for a few days, and then dry up without the infection going farther; yet those very persons, inoculated afresh, have, at the proper time, had the small-pox fever and eruption; although the fact related by Mr. Dawson serves to prove, that the ichorous discharge from the first incision, in

* The reader will find an abstract of Mr. Dawson's paper in the next article.

those thoſe patients, would have been capable of communicating the variolous infection to other perſons.

Nurſes, who ſuckle children ill of ſmall pox, frequently have a few puſtules on their breaſts and arms without any previous fever; and any body who attends cloſely to, and handles patients in that diſtemper, will be liable to have puſtules in the ſame manner. This has more than once happened to myſelf ſince the year 1745, when I had the ſmall pox in the natural way; and that ſuch local affection is truly variolous, the following experiment puts beyond a doubt.

In July 1768, ſix valuable negroes were inoculated from matter taken from a patient in the natural ſmall pox; but their arms dried up about the 6th day. As many negroes on the ſame eſtate had that diſorder, there was danger of their catching it in the natural way. They were therefore ſent to my houſe, to be again inoculated, and to ſtay till the iſſue was certain. At that very time I had a large variolous puſtule on my left thumb, of ſeven days ſtanding. No other infection being at hand, I inoculated the ſix negroe men from this poſtule. The infection took place; they had the variolous variolous fever on the feventh and eighth days, and the eruption appeared in the ufual manner. Two of thefe men had about five hundred puftules, the other four had the diforder more mildly. They returned home quite recovered in fixteen days from this laft inoculation.

*London,*
Jan. 10, 1786.

